# A cross-sectional study to evaluate access to antenatal care services in Twifo Hemang Lower Denkyira district of Ghana

**DOI:** 10.1371/journal.pone.0359892

**Published:** 2026-10-07

**Authors:** John Hammond, Silas Adjei-Gyamfi, Doreen Brew Daniels, Godfred Kwabena Sarpong, Hirotsugu Aiga, Tsunoneri Aoki

**Affiliations:** 1 Central Regional Health Directorate, Ghana Health Service, Cape Coast, Central Region, Ghana; 2 School of Tropical Medicine and Global Health, Nagasaki University, Nagasaki, Japan; 3 Faculty of Health and Environmental Sciences, Auckland University of Technology, Auckland, New Zealand; 4 Savelugu Municipal Hospital, Ghana Health Service, Savelugu, Northern Region, Ghana; 5 Elmina Polyclinic, Ghana Health Service, Elmina, Central Region, Ghana; 6 Institute of Tropical Medicine (NEKKEN), Nagasaki University, Nagasaki, Japan; 7 Inter-Faculty Initiative for Planetary Health (IIPH), Nagasaki University, Nagasaki, Japan; 8 Japan Human Development Department, International Cooperation Agency (JICA), Tokyo, Japan; 9 Japan International Cooperation Agency (JICA) Palau Country Office, Koror, Palau; Simon Diedong Dombo University of Business and Integrated Development Studies, GHANA

## Abstract

**Background:**

Antenatal care (ANC) supports skilled delivery and maternal survival, but evidence on how Ghana’s Community-Based Health Planning and Services (CHPS) policy influences ANC access is limited. This study assessed factors associated with ANC access in the Twifo Hemang Lower Denkyira District, Central Region, Ghana.

**Methods:**

In a cross-sectional study, we examined 310 women aged 15–49 years, having children less than 12 months, using a structured questionnaire administered through interviews. ANC initiation was defined as early when the first ANC visit occurred at 0–12 weeks’ gestation (first trimester) and late when it occurred at 13 weeks or later. We performed univariate and multivariate logistic regression analyses in Stata 17 and reported adjusted odds ratios (AORs) and 95% confidence intervals (CIs).

**Results:**

ANC attendance was 93.9%, and 69.1% of women initiated ANC early. Unmarried women (AOR = 0.125, 95%CI:0.012–0.926, p = 0.047) and women who delivered at home (AOR = 0.013, 95%CI:0.001–0.176, p = 0.001) had lower odds of attending at least one ANC visit. Late ANC initiation was associated with household size of 11 or more members (AOR = 3.848, 95%CI:1.914–6.211, p = 0.046), fewer than four ANC contacts (AOR = 6.332, 95% CI: 2.049–9.571, p = 0.001), and CHPS staff home visits (AOR = 1.813, 95%CI:1.014–3.243, p = 0.045). Higher monthly income was associated with lower odds of late ANC initiation (AOR = 0.123, 95%CI:0.024–0.630, p = 0.012). ANC and pregnancy knowledge, receiving care in a CHPS zone, and distance to a CHPS zone were not significantly associated with ANC attendance or timing after adjustment.

**Conclusions:**

ANC attendance and early initiation were relatively high. However, marital status, place of delivery, household size, income, ANC contacts, and CHPS home visits were associated with ANC access. Strengthening community-based and multisectoral interventions may improve timely ANC use and maternal health outcomes.

## Introduction

The international human rights law recognizes the right of women and adolescent girls to survive pregnancy and childbirth through the provision of appropriate sexual and reproductive health services [1]. According to the World Health Organization (WHO), access to adequate healthcare must be provided with sufficient and appropriate content [2]. However, globally, over 400 million people lack access to primary care [3], including pregnant women.

Antenatal care (ANC), which is the care provided by skilled healthcare providers to women during pregnancy for positive birth outcomes, forms an essential part of the reproductive, maternal, newborn, and child health (RMNCH) continuum of care. Typically, the care package include health-promotion, screening, and preventive services provided throughout pregnancy, including routine checks, supplementation, malaria prevention, HIV testing and counseling, and birth-preparedness counseling. The WHO 2016 model recommends at least eight contacts, beginning in the first trimester [4]. ANC has become a global priority over the past decade, intending to increase its coverage [5]; as such, between 2015 and 2022, about 88.8% of pregnant women in SSA had at least one ANC visit with a skilled care provider, although only 58.4% attained the recommended four or more visits [6]. Also, in sub-Saharan Africa (SSA), the proportion of women who had at least one ANC visit during pregnancy has improved from 69% in 2006 [7] to 78.5% in 2021 [8]. RMNCH services like ANC not only reduce maternal and child mortalities [9,10] but also prevent adverse birth outcomes like low birthweight and preterm births [11,12], especially in poorly resourced countries [4]. Early identification of high-risk pregnancies through ANC, together with the timely provision of appropriate care by skilled health personnel throughout the continuum of care, is critical to preventing maternal and newborn deaths. Effective ANC enables the early detection and management of pregnancy complications such as severe bleeding, infections, pre-eclampsia/eclampsia, and complications related to abortion, which together account for nearly 80% of maternal deaths. Ensuring that women receive timely, quality care right from pregnancy and childbirth to the postnatal period remains one of the most effective strategies for saving the lives of women and their children [10,13–15].

Challenges that women face in accessing skilled care during pregnancy and childbirth in resource-poor settings are a significant contributor to maternal deaths [16]. Although SSA accounted for only 17% of global births, the region accounted for 57% of the estimated 358,000 maternal deaths that occurred worldwide due to pregnancy- and childbirth-related complications [17]. Most studies have attributed ANC accessibility to factors including geographic/availability barriers (distance to the nearest CHPS zone), affordability barriers (household income and out-of-pocket ANC payments), acceptability barriers (knowledge of ANC and pregnancy, socio-cultural beliefs), and utilization patterns (timing and frequency of ANC contacts), among others, as predictors of timely and continuous ANC attendance [9,18,19]. These inequities in ANC underscores the need to understand context-specific health-system responses such as Ghana’s CHPS policy.

Over the past two decades, Ghana has been implementing the community-based health planning and services (CHPS) policy, aiming to reduce access barriers to healthcare through the provision of primary health care (PHC) services at the community level. The primary strategy of the policy is the deployment of a Community Health Officer (CHO) (a Community Health Nurse (CHN) who has undergone in-service training and orientation) and has been posted to a clearly defined CHPS zone supported by the community leadership structure to plan and mobilize resources for service delivery [20–22]. As the country’s leading approach to community-focused service provision, the initiative has contributed to a significant drop in maternal and child deaths and the increasing performance of ANC and vaccination services, particularly in rural areas [23]. According to the 2017 Ghana Maternal Health Survey, a nationally representative survey, 2.3% of women nationally did not attend ANC at all before their most recent delivery [24], 22% of all deliveries were assisted by unskilled personnel [25], and only 9.9% of births occurred within 8 km of CHPS zones [26], and this could partly explain why Ghana did not meet the Millenium Development Goal’s target on maternal and infant health.

In the Central Region of Ghana, CHPS facilities which serve 52% of rural populations in the region, contribute to about 16% of all ANC registrants and 8% of skilled deliveries [27]. The Twifo Hemang Lower Denkyira (THLD) district is one of the rural districts with poor maternal health indicators, particularly low coverage in ANC registration, skilled delivery, PNC care, family planning services, and high teenage pregnancies, despite the increased investment in scaling up PHC services closer to the doorsteps of many more households and communities. This is reflected, for instance, in the district’s 2020 ANC coverage (56.4%) and skilled delivery coverage (32.5%), both of which were below the Central Region averages of 81% and approximately 66%, respectively [27], indicating a gap between ANC and skilled delivery, as seen in most SSA countries. There is a need to understand how pregnant women in rural settings interact with the healthcare system and what influences their access to ANC in the context of CHPS. Considering several pieces of evidence that make CHPS a very promising policy for the improvement of services, particularly for women, access to healthcare in the district remains poor. Evidence on access to ANC services provided through the CHPS strategy for women in the region and its impact on achieving quality maternal and child healthcare is limited. This study therefore aimed to explore factors that influence women’s access to ANC services provided through the CHPS strategy in the THLD district of the Central Region of Ghana.

## Materials and methods

### Study setting

The study was conducted at THLD district which is one of the districts among the 22 districts in the Central region of Ghana, with its administrative capital at Twifo Hemang. It stretches about 75 km from the regional capital, Cape Coast, and lies between latitudes 5^o^ 50’ N and longitude 1^o^ 50’ W, located in the north-western part of the region with a total landmass of 674 sq. km (Fig 1). The estimated population in 2020 was 67,341, of which 51% are females, with an estimated 4% (2,694) Women in Fertility Age (WIFA) and a population density of about 82 per square kilometer [28]. Whereas the number of women of reproductive age in 2020 was 16,073, the total ANC and postnatal visits were 7,502 and 1,324 respectively. THLD district is predominantly rural (86%) with 300 settlements and 84 clearly defined communities. Most of the remaining settlements are farmsteads, usually with a population below 500 people. The health delivery system in the district is made up of the orthodox and traditional systems, with the latter consisting of traditional birth attendants, herbalists, fetish priests, and spiritualists, and is thought to play a crucial role in the health-seeking behavior of most people in the district. Health delivery under the orthodox system in the district exists up to two levels (A and B) under the country’s PHC system, that is, the community and sub-district levels. It is divided into four sub-districts with no district hospital but has three health centres, one community clinic, and 13 functional CHPS zones [27] as shown in Fig 1.

**Fig 1:**
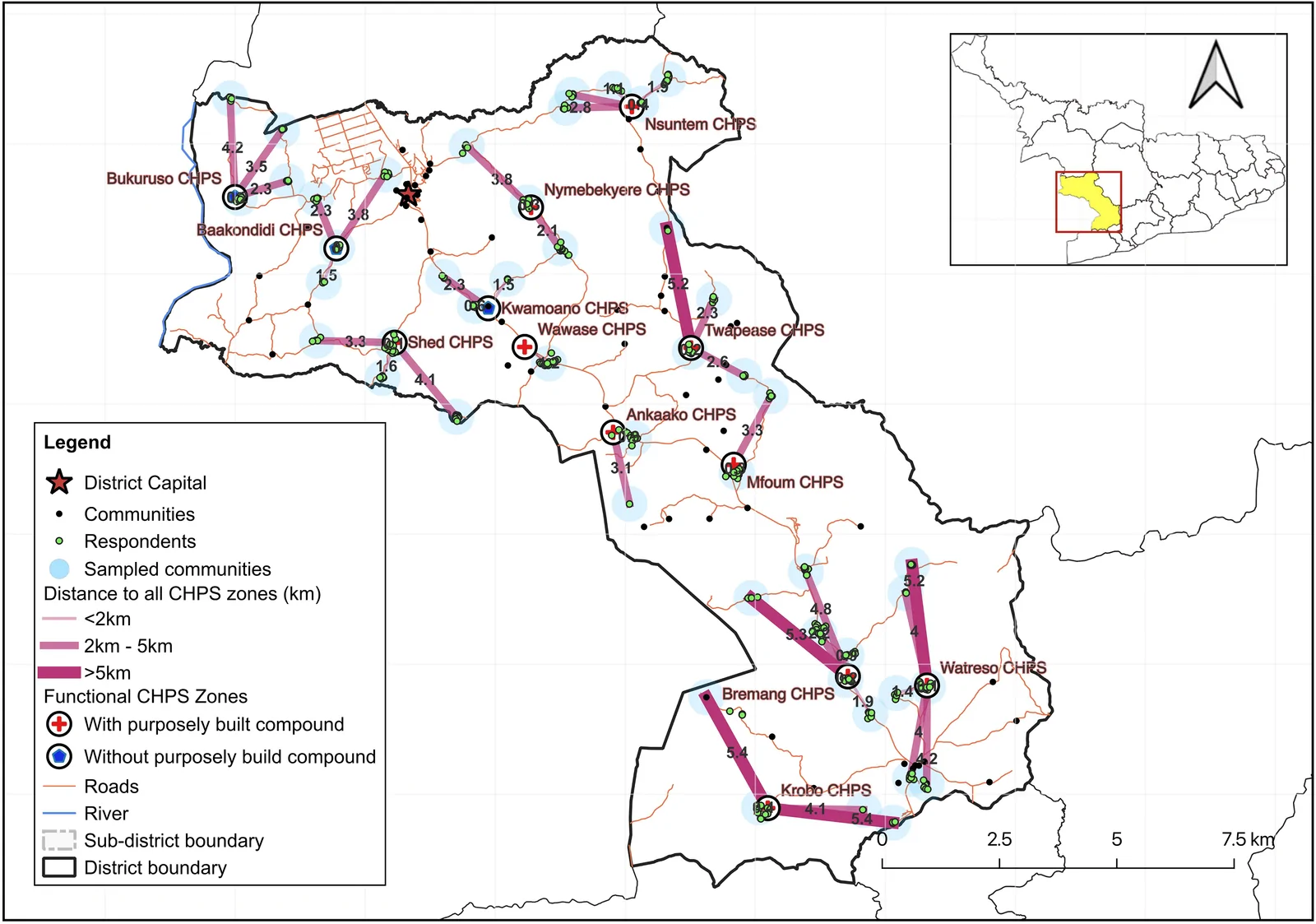
Map of the THLD district showing communities and measured distance to all CHPS zones.

### Study design and population

To explore access to ANC services in the THLD district of the Central Region under CHPS policy, we used a descriptive cross-sectional quantitative study design. This method allows simultaneous measurement of exposure and outcome status in a defined population and is well suited to estimating the prevalence of, and correlates associated with, ANC attendance and initiation timing in a population not previously characterized in this district [29,30]. This study targeted women of reproductive age (15–49 years) having at least one child aged 12 months or younger preceding data collection, living or residing within communities of all the demarcated CHPS zones of the district.

### Sample size

The sample size was determined using Cochran’s (1977) formula for estimating a sample size for a categorical outcome in a population whose exact size is not precisely known, which is appropriate given that the exact number of eligible mother–child pairs in the THLD district at the time of the study was not available [31,32]. The formula is expressed as N0 = [{(Zα/2)2(1–p)p}/d2], where “ N0” is the estimated sample size, “Zα” is the confidence level at 95% (standard value of 1.96), “p” is the estimated proportion of women accessing maternal healthcare services in CHPS zones in Ghana (76%) [18], and “d” is the level of precision/margin of error of 0.05. Hence, N0 = [{(1.96)² × (1–0.76) × 0.76}/(0.05)²] = 280.3 ≈ 280. With a minimum sample of 280, a non-response rate of 10% was added to attain an estimated final sample size of 310.

### Sampling method

During sampling, all 13 CHPS zones in the THLD district were selected for the study. We used the probability proportional to size (PPS) technique to determine sample sizes for each CHPS zone. This ensured each zone’s contribution to the final sample reflected its actual share of eligible mother–child pairs, preventing over-representation of smaller zones and under-representation of larger ones, and producing a self-weighting sample for CHPS zone-level population size. Using the vaccination records in the child welfare clinic register in each CHPS zone as the sampling frame [33], a total of 1751 eligible participants (children born under 12 months) were obtained. Thereafter, a simple random sampling method was used to select the required sample size of 310 from the CHPS zones and the selected children were directly linked to their mothers and sorted according to the community of residence. In total, 48 communities were obtained and the addresses (including telephone numbers) of the mothers were taken for identification at the community level. Within the community, research assistants were supported by the CHN/Os and the community volunteers to trace the mother-child pairs. The respondents were interviewed in their place of residence after booking an appointment with them and signing an informed consent and/or assent form. In the absence of a study respondent at the time of the visit, the research assistant rescheduled the interview to a later time. To maintain confidentiality, all personal identifiers such as addresses, and telephone numbers were stored separately from the research data in password-protected files accessible only to the research team. Each participant was assigned a unique identification code, and analyses were conducted using coded data rather than personal information. Hard-copy records were kept in locked cabinets, and no identifying details were disclosed in reports or publications.

### Data collection instrument

Data were collected using a structured, interviewer-administered questionnaire comprising five sections: (i) socio-demographic characteristics; (ii) socio-economic characteristics, including a household-asset module used to construct the wealth index; (iii) antenatal and obstetric history; (iv) knowledge of ANC and pregnancy (seven items, described under Measurement of study variables); and (v) CHPS location/distance characteristics, supplemented by a GPS-coordinate capture field. The questionnaire, adapted from instruments used in comparable rural Ghanaian and West African maternal-health studies [9,33,34], was developed in English before being translated into Fantse for administration.

The questionnaire was pretested among 31 mother-child pairs in Twifo Praso community, a neighboring community in Twifo Atti-Morkwa District, in January 2022. The pretest assessed question clarity, cultural appropriateness of the Fantse translation, question flow, logical flow, and average interview time. Following the pretest, 4 items were reworded for clarity, and 1 item was removed as redundant.

### Participant recruitment and rigor

Participant recruitment followed the process described under the data collection method below, with research assistants supported by CHNs/CHOs and community volunteers to trace and approach eligible mothers at their place of residence. All five research assistants held a minimum qualification of Bachelor’s degree in Nursing, Public Health, or Health Informatics and underwent 2 days of training prior to data collection, covering research ethics and informed consent/assent procedures, standardized questionnaire administration and probing techniques, use of the ODK mobile data-collection platform, and GPS coordinate capture. Three of the five research assistants were residents of, or had prior professional experience working within, THLD district, and all were fluent in Fantse and/or Twi.

### Data collection

The five trained research assistants conducted data collection from 15^th^ February to 15^th^ March 2022. Data was collected using a pretested standardized questionnaire with the aid of mobile data collection software (Open Data Kit Collect-version 2021.2.4). Moreover, two independent reseachers prevalidated the questionnaire (including knowledge related questions) and its content validity was confirmed by experts reviews and study authors. The information on socio-demographic, socioeconomic (household assets), antenatal, and CHPS location/distance characteristics as well as respondents’ knowledge of ANC and pregnancy were collected through face-to-face interviews with participants using the local dialect (Fantse and Twi). Other relevant information, including gestational age at first ANC visit, total number of ANC contacts completed, and any documented danger signs, was confirmed, where available, from the maternal and child health record books held by respondents. Before each session of the interview, global positioning system (GPS) coordinates were obtained from respondents which were used to estimate distances from the respondent’s place of residence to the nearest CHPS zone.

### Measurement of study variables

A description of the key variables is shown in Table 1. The outcome variables in the study were At least one ANC attendance (At least one ANC visit; No ANC visit) and time of ANC initiation categorized as Early initiation (first ANC ≤ 12 weeks of gestation) and Late initiation (first ANC ≥ 13 weeks of gestation). The exposure variables included all maternal background (sociodemographic, socioeconomic, and antenatal variables) and CHPS location/distance characteristics.

**Table 1:** Description of key study variables.

Variable	Definition and description	Variable type	Categories used
ANC attendance	The ability of respondents to make at least one ANC visit during pregnancy	Binary	At least one ANC visit; No ANC visit
ANC initiation	Timing of the first ANC visit before delivery of the youngest child ≤12 months and classified into Early initiation (0–12 weeks of gestation or first trimester) and late initiation (≥13 weeks of gestation or second and third trimester)	Binary	Early ANC initiation (first ANC ≤ 12 weeks of gestation); Late ANC initiation (first ANC ≥ 13 weeks of gestation)
Age group	Age at last birthday in years and categorised	Ordinal	15–19; 20–24; 25–29; 30–34; 35+
Marital status	Having customary or legal partner	Binary	Married; Not married
Religion	Religious affiliation of the respondent	Binary	Christianity; Islam
Ethnicity	Belonging to any tribal or ethnic group	Nominal	Fantse; Denkyira; Ewe; Akuapim; Others
Educational level	The highest level of education attained by respondents and partners	Nominal	No formal education; Primary; Secondary/ higher
Household/ family size	The number of people living within the household of respondents.	Ordinal	1–5; 6–10; 11+
Parity	The total number of children born by respondent	Ordinal	1; 2–4; 5+
Age of child	The age of the youngest child in completed months verified with the child vaccination records.	Ordinal	≤4; 5–8; 9–12
Employment	The status of occupation of the respondent and the partner	Binary	Unemployed; Employed
Household wealth index	Constructed using household items possessed by respondents and analyzed using principal component analysis before grouping them into terciles.	Nominal	Poor; Middle; Rich
Place of ANC attendance	Type of facility where respondent received ANC care during pregnancy.	Nominal	CHPS; Clinic; Health centre; Hospital/ Polyclinic
Frequency of ANC visit	Number of ANC visits before delivery and categorized into <4 and ≥4 visits.	Binary	<4; ≥ 4
Decision to attend ANC	Who decides for the respondent to attend or not to attend ANC during her pregnancy.	Nominal	Myself; Husband/ partner; Others
Knowledge	Participants’ level of knowledge about ANC and pregnancy and categorised into Inadequate and Adequate by assessing composited knowledge scores using a median cut-off point	Binary	Inadequate; Adequate
*CHPS functionality status	The state of functionality of the CHPS zone according to the Ghana Health Service CHPS implementation guideline (2016). A CHPS zone was classified as ‘uncompleted functional’ where community entry and staffing milestones had been achieved and a CHO was actively deployed and providing services, but the standard physical compound structure and/or logistical equipment remained incomplete or under construction, and ‘completed functional’ where both staffing and infrastructure milestones had been achieved [20,21]	Binary	Uncompleted; Completed
Distance to CHPS zone	The measured distance from participants’ place of residence to the nearest CHPS zones.	Binary	<5km; ≥ 5 km

### Data analysis

Data analysis was conducted using STATA version 17 (Stata Corporation, Texas, USA) at a significance of p < 0.05. Simple descriptive statistics were performed on categorical variables and summarized into frequency distribution and percentages. Means and standard deviations were calculated for continuous variables and were further categorized based on the existing literature. ANC attendance was coded as “0” for “No ANC attendance” and “1” for “ At least one ANC attendance”; while the timing of ANC initiation was also coded as “0” for “Early ANC initiation” and “1” for “Late ANC initiation”. Socioeconomic characteristics were measured with principal component analysis to construct household wealth terciles (poor, middle, and rich) using household assets owned by respondents [35]. Knowledge was assessed using a composite score derived from seven items adapted from the Ghana Health Service and WHO guidelines for essential ANC content: (i) who is supposed to attend ANC; (ii) the best time to start ANC; (iii) the recommended number of ANC visits for a normal pregnancy; (iv) the purpose of ANC; (v) the benefit of ANC for pregnant women; (vi) danger signs during pregnancy requiring skilled care and (vii) the source of the respondent’s information about ANC. Each correct response scored 1 point (≥4 of 7) were classified as having ‘adequate knowledge,’ following the median-based classification approach used in comparable studies [11,33,34]. Moreover, the knowledge score demonstrated good reliability with a Cronbach’s alpha coefficient of 0.84. All GPS coordinates collected from participants were processed in QGIS using the Euclidean distance method to calculate the distance from each respondent’s residence to the CHPS zones [36,37].

Bivariate analyses (Chi-square/Fisher’s exact test) were used to explore the association between the outcome variables and all exposure variables, comprising sociodemographic, socioeconomic, antenatal/maternal, and CHPS location/distance characteristics (Tables 2–4). We then conducted simple logistic regression analyses to confirm the bivariate associations. Predictor variables demonstrating statistical significance at a p-value of <0.05 in the bivariate association were forwarded into the multivariate logistic regression model after controlling for multicollinearity. During multicollinearity testing via variance inflation factor (VIF), the variables with a VIF less than 5 were chosen for the final logistic analyses [12]. While a more liberal univariate screening threshold (p < 0.20–0.25) is sometimes recommended to avoid prematurely excluding potentially important predictors [38], we retained the conventional p < 0.05 threshold in view of the case-to-variable ratio constraints imposed by our sample size and the associated risk of model overfitting and instability [39]. Multivariate logistic regression was used to identify factors for at least one ANC attendance or late ANC initiation and the analysis results were reported as odds ratios with its corresponding 95% confidence interval.

**Table 2:** Sociodemographic characteristics of respondents.

		Bivariate analysis					
Variables	Total (%)	ANC attendance (%)		p-value^a^	ANC Initiation (%)		p-value^a^
		No (n = 19)	Yes (n = 291)		Early (n = 201)	Late (n = 90)	
**Age group** (mean:27.6; sd:6.9)							
15–19 years	47 (15.2)	1 (5.3)	46 (15.8)	0.602	25 (12.4)	21 (23.3)	0.150
20–24 years	65 (21.0)	5 (26.3)	60 (20.6)		40 (19.9)	20 (22.2)	
25–29 years	69 (22.3)	5 (26.3)	64 (22.0)		48 (23.9)	16 (17.8)	
30–34 years	75 (24.2)	6 (31.6)	69 (23.7)		51 (25.4)	18 (20)	
35 + years	54 (17.4)	2 (10.5)	52 (17.9)		37 (18.4)	15 (16.7)	
**Marital status**							
Not married	68 (21.9)	8 (42.1)	60 (20.6)	0.028^*^	38 (18.9)	22 (24.4)	0.280
Married	242 (78.1)	11 (57.9)	231 (79.4)		163 (81.1)	68 (75.6)	
**Religious affiliation**							
Christianity	296 (95.5)	17 (89.5)	279 (95.9)	0.193	193 (96.0)	86 (95.6)	0.854
Islam	14 (4.5)	2 (10.5)	12 (4.1)		8 (4.0)	4 (4.4)	
**Ethnicity**							
Akuapim	147 (47.4)	2 (10.5)	19 (6.5)	0.753	95 (47.3)	42 (46.7)	0.623
Denkyira	73 (23.6)	3 (15.8)	70 (24.1)		52 (25.9)	18 (20.0)	
Ewe	36 (11.6)	3 (15.8)	33 (11.3)		23 (11.4)	10 (11.1)	
Fantse	21 (6.8)	10 (52.6)	137 (47.1)		12 (6.0)	7 (7.8)	
Others	33 (10.7)	1 (5.3)	32 (11.0)		19 (9.4)	13 (14.4)	
**Mother’s educational level**							
No formal education	40 (12.9)	5 (26.2)	35 (12.0)	0.003^*^	22 (11.0)	13 (14.4)	0.196
Primary	49 (15.8)	7 (36.9)	42 (14.4)		25 (12.4)	17 (18.9)	
Secondary/Higher	221 (71.3)	7 (36.9)	214 (73.6)		154 (76.6)	60 (66.7)	
**Partner’s educational level**							
No formal education	43 (13.9)	6 (31.6)	37 (12.7)	0.043^*^	22 (11.0)	15 (16.7)	0.374
Primary	20 (6.5)	2 (10.5)	18 (6.2)		12 (6.0)	6 (6.7)	
Secondary/Higher	247 (79.7)	11 (57.9)	236 (81.1)		167 (83.0)	69 (76.6)	
**Household size** (mean:6.2; sd:2.5)							
1–5 people	133 (42.9)	7 (36.8)	126 (43.3)	0.348	93 (46.3)	33 (36.7)	0.030^*^
6–10 people	164 (52.9)	10 (52.6)	154 (52.9)		104 (51.7)	50 (55.5)	
11 + people	13 (4.2)	2 (10.6)	11 (3.8)		4 (2.0)	7 (7.8)	
**Parity** (mean:2.3; sd:1.4)							
1	126 (40.6)	9 (47.4)	117 (40.2)	0.102	78 (38.8)	39 (43.3)	0.289
2-4	143 (46.1)	5 (26.3)	138 (47.4)		101 (50.2)	37 (41.1)	
5+	41 (13.3)	5 (26.3)	36 (12.4)		22 (11.0)	14 (15.6)	
**Age of child** (mean:6.7; sd:3.5)							
≤ 4 months	101 (32.6)	7 (36.8)	94 (32.3)	0.905	66 (32.8)	28 (31.1)	0.255
5–8 months	99 (31.9)	6 (31.6)	93 (32.0)		69 (34.4)	24 (26.7)	
9–12 months	110 (35.3)	6 (31.6)	104 (35.7)		66 (32.8)	38 (42.2)	
**Mother’s occupation**							
Unemployed	60 (19.4)	3 (15.8)	57 (19.6)	0.644	36 (17.9)	21 (23.2)	0.217
Farmer	122 (39.4)	11 (57.8)	111 (38.1)		75 (37.3)	36 (40.0)	
Trader	66 (21.3)	3 (15.8)	63 (21.5)		48 (23.8)	15 (16.7)	
Seamstress	20 (6.5)	0 (0.0)	20 (6.9)		13 (6.5)	7 (7.8)	
Hairdresser	17 (5.5)	1 (5.3)	16 (5.5)		10 (5.0)	6 (6.7)	
Public/civil servant	11 (3.5)	0 (0.0)	11 (3.9)		11 (5.5)	0 (0.0)	
Others	14 (4.5)	1 (5.3)	13 (4.5)		8 (4.0)	5 (5.6)	
**Partner’s main occupation**							
Unemployed	1 (0.3)	0 (0.00)	1 (0.3)	0.298	1 (0.5)	0 (0.0)	0.933
Farmer	149 (48.1)	13 (68.4)	136 (46.7)		93 (46.27)	43 (47.7)	
Artisan	57 (18.4)	4 (21.1)	53 (18.2)		38 (18.91)	15 (16.6)	
Driver	43 (13.9)	0 (0.0)	43 (14.8)		29 (14.43)	14 (15.6)	
Trader	24 (7.7)	2 (10.5)	22 (7.6)		14 (6.97)	8 (8.9)	
Public/civil servant	22 (7.1)	0 (0.0)	22 (7.6)		17 (8.46)	5 (5.6)	
Others	14 (4.5)	0 (0.0)	14 (4.8)		9 (4.48)	5 (5.6)	

* p-value < 0.05, ^a^Chi-square/Fisher’s exact test.

**Table 3:** Socioeconomic and antenatal characteristics of respondents.

		Bivariate analysis					
Variables	Total (%)	ANC attendance (%)		p-value^a^	ANC Initiation (%)		p-value^a^
		No (n=19)	Yes (n=291)		Early (n=201)	Late (n=90)	
**Mother’s average monthly income**							
No income	60 (19.4)	3 (15.8)	57 (19.5)	0.517	36 (17.9)	21 (23.4)	0.017^*^
<GHc 100.0 (<$9.0)	21(6.8)	1 (5.3)	20 (6.9)		10 (5.0)	10 (11.1)	
GHc 100-299 ($9-33)	40 (12.9)	2 (10.5)	38 (13.1)		28 (13.9)	10 (11.1)	
GHc 300-499 ($34-55)	35 (11.3)	4 (21.0)	31 (10.7)		23 (11.5)	8 (8.9)	
GHc 500-1000($56-111)	30 (9.7)	0 (0.0)	30 (10.3)		28 (13.9)	2 (2.2)	
Don’t know	124 (40.0)	9 (47.4)	115 (39.5)		76 (37.8)	39 (43.3)	
**Mother’s health insurance status**							
No	26 (8.4)	4 (21.1)	22 (7.6)	0.040^*^	13 (6.5)	9 (10.0)	0.292
Yes	284 (91.6)	15 (78.9)	269 (92.4)		188 (93.5)	81 (90.0)	
**Household wealth index**							
Poor	131 (42.3)	15 (78.9)	116 (39.8)	0.003^*^	75 (37.31)	41 (45.6)	0.029^*^
Middle	78 (25.2)	3 (15.8)	75 (25.8)		47 (23.38)	28 (31.1)	
Rich	101 (32.5)	1 (5.3)	100 (34.4)		79 (39.3)	21 (23.3)	
**Knowledge about ANC and pregnancy**							
Inadequate	11 (3.5)	10 (52.6)	1 (0.3)	<0.001^*^	1 (0.5)	0 (0.0)	0.503
Adequate	299 (96.5)	9 (47.4)	290 (99.7)		200 (99.5)	90 (100)	
**Place of ANC**							
CHPS	209 (71.8)	–	209 (71.8)	–	148 (73.6)	61 (67.8)	0.409
Clinic	5 (1.7)	–	5 (1.7)		2 (1)	3 (3.3)	
Health centre	69 (23.7)	–	69 (23.7)		45 (22.4)	24 (26.7)	
Hospital/ Polyclinic	8 (2.8)	–	8 (2.8)		6 (3.0)	2 (2.2)	
**Frequency of ANC visit**							
<4 visits	20 (6.9)	19 (100.0)	20 (6.9)	–	6 (3.0)	14 (15.6)	<0.001^*^
≥4 visits	271 (93.1)		271 (93.1)		195 (97.0)	76 (84.4)	
**Accompanied for ANC visit (at least one)**							
No one (Alone)	198 (68.0)	–	198 (68.0)	–	139 (69.1)	59 (65.6)	0.543
Husband/partner/ other	93 (32.0)	–	93 (32.0)		62 (30.9)	31 (34.4)	
**ANC service provider**							
CHN/CHO	7 (2.4)	–	7 (2.4)	–	6 (3.0)	1 (1.1)	0.335
Midwife	284 (97.6)	–	284 (97.6)		195 (97.0)	89 (98.9)	
**Pay for ANC service**							
No	193 (66.3)	–	193 (66.3)	–	131 (65.2)	62 (68.9)	0.535
Yes	98 (33.7)	–	98 (33.7)		70 (34.8)	28 (31.1)	
**Average amount spent per ANC visit**							
GHc 1.0-5.0 ($0.1-0.5)	26 (26.5)	–	26 (26.5)	–	17 (24.29)	9 (32.1)	0.445
GHc 6.0-10.0 ($0.6-1.1)	24 (24.5)	–	24 (24.5)		17 (24.29)	7 (25)	
GHc 11.0-15.0 ($1.2-1.7)	11 (11.2)	–	11 (11.2)		6 (8.57)	5 (17.9)	
GHc 16.0-20.0 ($1.8-2.2)	20 (20.4)	–	20 (20.4)		16 (22.86)	4 (14.3)	
>GHc 20.0 (>$2.2)	17 (17.4)	–	17 (17.4)		14 (20)	3 (10.7)	
**Decision maker before attending ANC**							
Self	240 (77.4)	17 (89.5)	223 (76.6)	0.195	151 (75.1)	72 (80.0)	0.364
Husband/partner	70 (12.6)	2 (10.5)	68 (23.4)		50 (24.9)	18 (20.0)	
**Place of delivery**							
Home	61 (19.7)	15 (78.9)	46 (15.8)	<0.001^*^	25 (12.4)	21 (23.3)	0.119
Health facility	249 (80.3)	4 (21.1)	245 (84.2)		176 (87.6)	69 (76.7)	
**Received home visit from CHPS zone staff**							
No	202 (65.2)	7 (36.8)	195 (67.0)	0.007^*^	142 (70.6)	53 (58.9)	0.039^*^
Yes	108 (34.8)	12 (63.2)	96 (33.0)		59 (29.4)	37 (41.1)	

* p-value < 0.05, ^**a**^Chi-square/Fisher’s exact test.

**Table 4:** Distance and location of CHPS zones characteristics.

		Bivariate analysis					
Variables	Total (%)	ANC attendance (%)		p-value^a^	ANC Initiation (%)		p-value^a^
		No (n = 19)	Yes (n = 291)		Early (n = 201)	Late (n = 90)	
**Health facility in community**							
No	163 (52.6)	18 (94.7)	145 (49.8)	<0.001^*^	98 (48.8)	47 (52.2)	0.585
Yes	147 (47.4)	1 (5.3)	146 (50.2)		103 (51.2)	43 (47.8)	
**Type of nearest health facility in community**							
CHPS	250 (80.7)	18 (94.7)	232 (79.7)	0.268	155 (77.1)	77 (85.6)	0.251
Health centre	10 (3.2)	1 (5.3)	49 (16.8)		38 (18.9)	11 (12.2)	
Clinic	50 (16.1)	0 (0.00)	10 (3.5)		8 (4.0)	2 (2.2)	
**CHPS zone functionality status**							
Uncompleted functional	48 (15.5)	7 (36.8)	41 (14.1)	0.008^*^	20 (10.0)	21 (23.3)	0.002^*^
Completed functional	262 (84.5)	12 (63.2)	250 (85.9)		181 (90.0)	69 (76.7)	
**Average travel time to nearest CHPS zone (one-way)**							
≤ 30 min	253 (81.6)	13 (68.4)	240 (82.5)	0.126	171 (85.1)	69 (76.7)	0.081
> 30 min	57 (18.4)	6 (31.6)	51 (17.5)		30 (14.9)	21 (23.3)	
**Means of transport to nearest CHPS zone**							
Taxi/bus	58 (18.7)	16 (84.2)	264 (90.7)	0.593	185 (92.0)	79 (87.8)	0.342
Motorbike/tricycle	108 (34.8)	3 (15.8)	26 (8.9)		15 (7.5)	11 (12.2)	
Walk	144 (46.5)	0 (0.0)	1 (0.34)		1 (0.5)	0 (0.0)	
**Average transportation cost per visit**							
GHc 1–10.0 ($0.1–1.1)	280 (90.3)	16 (84.2)	264 (90.7)	0.593	185 (92.0)	79 (87.8)	0.342
GHc 11–20.0 ($1.2–2.2)	29 (9.4)	3 (15.8)	26 (8.9)		15 (7.5)	11 (12.2)	
> GHc 20.0 (>$2.2)	1 (0.3)	0 (0.0)	1 (0.4)		1 (0.50)	0 (0.0)	
**Condition of road to health facility**							
No road/Footpath	9 (2.9)	2 (10.5)	7 (2.4)	0.212	6 (3.0)	1 (1.1)	0.233
Feeder/dusty road	174 (56.1)	11 (57.9)	163 (56.0)		110 (54.7)	53 (58.9)	
Pothole riddled road	23 (7.4)	1 (5.3)	22 (7.6)		12 (6.0)	10 (11.1)	
Smooth tarred road	104 (33.6)	5 (26.3)	99 (34.0)		73 (36.3)	26 (28.9)	
**Distance to nearest functional CHPS zones** (mean:2.0; sd:1.5)							
≥ 5 km	60 (19.3)	7 (36.8)	53 (18.2)	0.046^*^	29 (14.4)	24 (26.7)	0.012^*^
< 5km	250 (80.7)	12 (63.2)	238 (81.8)		172 (85.6)	66 (73.3)	

* p-value < 0.05, ^**a**^Chi-square/Fisher’s exact test.

### Ethical declarations

Ethical approval was sought from the Nagasaki University Ethics Review Committee and Ghana Health Service Ethics Review Committee with protocol approval numbers NU_TMGH_2021_195_1 and GHS-ERC: 023/01/22, respectively. Additionally, written permission was obtained from the Central Regional and THLD District Health Directorates. Opinion leaders in the selected communities were informed about the presence of the research team. Written informed consent was obtained from all participants aged 18 years and older. For participants who were themselves minors (aged 15–17 years), written informed assent was obtained from the minor participant together with written informed consent from her parent, guardian, or legal representative.

## Results

### Sociodemographic characteristics of respondents

Of the 310 recruited mother-child dyads who fully responded to the study, 24.2% were between the ages of 30–34 years, with a mean(±sd) age of 27.6(±6.9) years. Most of the respondents (78.1%) were married, 80.6% engaged in some form of occupation, with almost all (95.5%) identifying as Christians. Nearly half (47.4%) belonged to the Fantse ethnic group, with those having 2–4 children being 46.1%. Majority of the respondents (87.1%) and their partners (86.1%) had attained some form of formal education with secondary/higher as their highest educational level. About 52.9% of the respondents had a household size of 6–10 people – while 35.5% indicated their youngest children were between the ages of 9–12 months (Table 2).

### Socioeconomic and antenatal characteristics of respondents

Most of the respondents were enrolled in the national health insurance scheme (91.6%) and had a higher knowledge level about ANC and pregnancy (96.5%) while 42.3% of them were classified as poor based on their household wealth index. Despite the free maternal healthcare policy in Ghana, all the women claimed they paid some money for receiving ANC services from the health facilities, with 26.5% of them paying between Ghc 1.0–5.0 (USD 0.1–0.5).

ANC coverage was nearly universal (93.9%) with most of them accessing ANC service from the CHPS zones (71.8%). In addition, 69.1% of the women initiated ANC services at an early stage of pregnancy (thus within the first trimester), while 12.6% of them made fewer than four visits before delivery. The decision to attend ANC was mainly made by most women themselves (77.4%), and almost one-third of the women were escorted by their partners (32.0%). Although 34.8% of the respondents received home visits from CHPS zone staff, the prevalence of home delivery was 19.7% (Table 3).

### Distance and location of CHPS zones

The distance and location of CHPS zones (health facilities) providing for ANC services is described in Table 4. More than half (52.6%) of the respondents resided in communities without health facilities, and 80.7% reported that the CHPS compound was the nearest health facility to them. However, 15.5% of the nearest CHPS zones were incomplete functional zones. About 81.6% of the women travel for less than 30 minutes before getting to the nearest CHPS zones, with their primary mode of transport being either walking (46.5%) or using commercial motorbikes (34.8%). Also, 90.3% of the women spent between GHc 1.0–10.0 (USD 0.1–1.1) to and from the nearest health facility per ANC visit on roads described by most as feeder/dusty (56.1%). Distance measured from the place of residence showed that about 80.7% of the women traveled less than five kilometers to the nearest functional CHPS zone (Fig 1).

### Bivariate association of antenatal accessibility with background variables

As displayed in Tables 2, 3 and 4, marital status (p = 0.028), mother’s educational level (p = 0.003), partner’s educational level (p = 0.043), mother’s health insurance status (p = 0.040), household wealth index (p = 0.003), knowledge about ANC and pregnancy (p < 0.001), place of delivery (p < 0.001), receiving home visit from CHPS zone staff (p = 0.007), health facility in the community (p < 0.001), CHPS zone functionality status (p = 0.008), and distance to nearest functional CHPS zones (p = 0.046) exhibited bivariate correlation with at least one ANC attendance. Again, variables that showed significant association with the late ANC initiation at the bivariate level included household (family) size (p = 0.030), mother’s average monthly income (p = 0.017), household wealth index (p = 0.029), frequency of ANC visits (p < 0.001), receiving home visit(s) from CHPS zone staff (p = 0.049), CHPS zone functionality status (p = 0.002), and distance to nearest functional CHPS zones (p = 0.012).

### Multivariate analysis on associated factors for antenatal accessibility

After registering no multicollinearity issues among the eleven and seven predictor variables that showed significant bivariate association with at least one ANC attendance and late ANC initiation respectively, two (marital status, place of delivery) and four (household size, mother’s average monthly income, frequency of ANC visits, receiving home visit from CHPS zone staff) predictor variables respectively remained statistically significant at the multivariate analysis level (Table 5).

**Table 5:** Logistic regression on associated factors for antenatal accessibility.

Logistic regression model	COR (95% CI)	p-value	AOR (95% CI)	p-value
***Logistic regression for ‘At least one ANC attendance’***				
**Marital status**				
Not married	0.357 (0.138 - 0.927)	0.034^*^	**0.125 (0.012 - 0.926)**	**0.047** ^*^
Married			Reference	
**Mother’s educational level**				
No formal education	0.229 (0.069 - 0.762)	0.016^*^	2.474 (0.081 - 75.18)	0.603
Primary	0.196 (0.065 - 0.589)	0.004^*^	2.325 (0.134 - 40.48)	0.563
Secondary/Higher			Reference	
**Partner’s educational level**				
No formal education	0.287 (0.100 - 0.824)	0.020^*^	0.445 (0.158 - 12.56)	0.635
Primary	0.419 (0.086 - 2.039)	0.282	0.922 (0.051 - 16.60)	0.956
Secondary/Higher			Reference	
**Mother’s health insurance status**				
No	0.307 (0.093 - 0.993)	0.041^*^	0.962 (0.068 - 13.54)	0.977
Yes			Reference	
**Household wealth index**				
Poor	0.077 (0.010 - 0.596)	0.014^*^	0.001 (0.0003 - 1.250)	0.934
Middle	0.250 (0.025 - 2.451)	0.234	0.009 (0.001 - 2.201)	0.992
Rich			Reference	
**Knowledge about ANC and pregnancy**				
Inadequate	0.003 (0.001 - 0.027)	<0.001^*^	0.006 (0.0001 - 1.152)	0.989
Adequate			Reference	
**Place of delivery**				
Home	0.050 (0.016 - 0.158)	<0.001^*^	**0.013 (0.001 - 0.176)**	**0.001** ^*^
Health facility			Reference	
**Received home visit from CHPS zone staff**				
Yes	0.287 (0.110 - 0.753)	0.011^*^	0.873 (0.281 - 12.49)	0.517
No			Reference	
**Health facility in community**				
No	0.055 (0.007 - 0.419)	0.005^*^	0.171 (0.013 - 2.302)	0.183
Yes			Reference	
**CHPS zone functionality status**				
Uncompleted functional	0.281 (0.105 - 0.756)	0.012^*^	0.203 (0.017 - 2.409)	0.206
Completed functional			Reference	
**Distance to nearest functional CHPS zones**				
≥ 5 km	0.382 (0.143 - 0.902)	0.044^*^	0.664 (0.068 - 6.441)	0.724
< 5km			Reference	
***Regression model fit***	*Nagelkerke R*^*2*^ *= 0.690; p < 0.001* *Hosmer-Lemeshow test: χ² = 5.89; df = 142;* p = *1.000*			
***Logistic regression for ‘Late ANC initiation’***				
**Family size**				
1–5 people	0.738 (0.438 - 1.243)	0.253	0.956 (0.537 - 1.703)	0.878
6–10 people			Reference	
11 + people	3.640 (1.018 - 5.011)	0.047^*^	**3.848 (1.914 - 6.211)**	**0.046** ^*^
**Mother’s average monthly income**				
No income			Reference	
< GHc 100.0 (<$9.0)	1.714 (0.613 - 4.794)	0.304	1. 227 (0.387 - 3.889)	0.728
GHc 100–299 ($9–33)	0.612 (0.249 - 1.507)	0.286	0.641 (0.242 - 1.697)	0.371
GHc 300–499 ($34–55)	0.974 (0.330 - 2.871)	0.295	0.617 (0.222 - 1.713)	0.354
GHc 500–1000 ($56–111)	0.122 (0.026 - 0.567)	0.007^*^	**0.123 (0.024 - 0.630)**	**0.012** ^*^
Don’t know	0.880 (0.454 - 1.706)	0.704	0.795 (0.387 - 1.633)	0.533
**Household wealth index**				
Poor	2.057 (1.113 - 3.798)	0.021^*^	1.993 (0.989 - 3.947)	0.058
Middle	2.241 (1.145 - 4.384)	0.018^*^	2.032 (0.971 - 4.248)	0.060
Rich			Reference	
**Frequency of ANC visit**				
< 4 visits	6.987 (2.219 - 8.151)	<0.001^*^	**6.332 (2.049 - 9.571)**	**0.001** ^*^
≥ 4 visits			Reference	
**Received home visit from CHPS zone staff**				
Yes	1.680 (1.001 - 2.821)	0.040^*^	**1.813 (1.014 - 3.243)**	**0.045** ^*^
No			Reference	
**CHPS zone functionality status**				
Uncompleted functional	2.754 (1.406 - 5.395)	0.003^*^	1.647 (0.693 - 3.912)	0.259
Completed functional			Reference	
**Distance to nearest functional CHPS zones**				
≥ 5 km	2.157 (1.171 - 3.972)	0.014^*^	1.520 (0.691 - 3.341)	0.297
< 5km			Reference	
***Regression model fit***	*Nagelkerke R*^*2*^ *= 0.732; p < 0.001* *Hosmer-Lemeshow test: χ² = 4.92; df = 118;* p = *0.859*			

* p-value < 0.05.

The findings revealed that unmarried women were 98.7% (=[1–0.013]x100) less likely to attend ANC services compared to married ones (AOR = 0.013; 95%CI = 0.001–0.176; p = 0.001). Women who delivered at home had lower odds of attending ANC during pregnancy than those who delivered in a health facility (AOR = 0.125; 95%CI = 0.012–0.926; p = 0.047). On the other hand, women with a larger household size of more than 11 people were 3.848 times more likely to initiate late ANC services than their counterparts (AOR = 3.848; 95%CI = 1.914–6.211; p = 0.046). The likelihood of a pregnant woman who made less than four ANC visits to initiate late ANC services is 6.332 times higher than those who made at least four ANC visits during pregnancy (AOR = 6.332; 95%CI = 2.049–9.571; p = 0.001). Additionally, pregnant women who received home visits by CHPS staff had 1.813 times increased odds of late ANC initiation compared to those who did not receive home visit sessions (AOR = 1.813; 95%CI = 1.014–3.243; p = 0.045). Finally, compared to women who had no income, those whose average monthly income ranged between GHc 500–1000 (USD 56–111) were 87.7% (=[1–0.123]x100) less likely to initiate ANC services in the second or third trimester of pregnancy (AOR 0.123–95%CI = 0.024–0.630; p = 0.012).

## Discussion

The study examined factors that influence access to ANC services in rural communities within the THLD district of the Central Region of Ghana in the context of CHPS policy implementation. Among 310 mother-child pairs, most respondents were married, employed, Christian, well-educated, and lived in households of 6–10 people. Nearly all had health insurance and adequate ANC knowledge, though many were poor and all paid some ANC costs despite free care policy. ANC coverage was 93.9%, with 69.1% initiating early and most attending via CHPS zones close to home. Bivariate analysis linked several sociodemographic, socioeconomic, and geographic factors to ANC outcomes. However, multivariate analysis showed only marital status and delivery location predicted ANC attendance, while household size, visit frequency, CHPS home visits, and income predicted late ANC initiation.

Findings from the study showed that 93.9% of the respondents had at least one ANC service from a skilled provider, with 69.1% initiating their first visit within the first trimester of pregnancy. This is higher than the regional ANC coverage and proportion of first-trimester visits of 81% and 53% respectively [27] but agrees with the general high coverage in Ghana as reported by several studies [24,40]. In contrast, other studies in Ghana [41] and Rwanda [42] reported only 43.1% and 25% of early ANC initiation respectively. In our study, 93% of the women also made four or more ANC visits before delivery which is higher than the Ghana national average of 89% [24] and rates in other developing countries [33,43,44]. This is an indication of how the interest of Ghanaian women has developed in the benefits of attending ANC, which could be attributed to the heightened efforts to expand health facility coverage to improve access to healthcare, especially in bringing quality healthcare services close to the people [45].

Marital status offered significant protection for ANC attendance, as unmarried women had reduced odds of making at least one ANC visit. This finding is consistent with many other studies conducted in Ghana [9,34,46,47], and Kenya [44], but contradicts the study results from Ethiopia [48,49]. The possible reason for this finding could be attributed to society’s values on marriage and pregnancy. For instance, in most rural Ghanaian communities, unmarried women are not to engage in sexual acts [9]. In a situation when pregnancy occurs, most of these women tend to hide their pregnancies until it is obvious to avoid being stigmatized and ostracized by society, which could contribute to reducing their ANC contacts or failing to attend ANC at all [34,44,50,51]. On the other hand, married women are seen to have a higher possibility of receiving support from their partners (husbands) during pregnancy [9] which increases their chances of higher ANC visits.

The place of delivery had a significant influence on ANC attendance, as women who delivered at home were less likely to make at least one ANC visit. This study’s results agree with similar studies conducted in most developing countries [41,52,53]. In Ghana, there is increased awareness creation (via health education, and counseling) on services for pregnant women, free maternal healthcare under the NHIS, as well as the expansion of CHPS, which is helping to bridge the access gap for vulnerable populations [9,22]. Notwithstanding, the perceived mistreatment of pregnant women by healthcare providers [54,55] could be responsible for the home deliveries and low ANC visits. It is also worth noting that, despite the availability of at least one midwife to offer skilled care for pregnant women in almost all the CHPS zones in the district, most women are demotivated to visit ANC for service due to some cultural and personal beliefs such as the fear of spiritual attack (e.g., witchcraft or “evil eye” known as Asram). This leads women to conceal the pregnancy in the early stages as well as the preference for traditional healers/birth attendants and use of herbal preparations due to familiarity [56,57].

Household size of more than 11 people was a significant correlate of late ANC initiation. In congruence with similar studies across the globe [58–61], having a larger household size is a strong predictor of late ANC initiation. Our study results contradict findings in Ethiopia [62] and Northern Ghana [51], which concluded that women with 4–9 living children were 3.5 times more likely to initiate ANC early. Household sizes in rural communities are mostly larger than those in urban communities [24], as more than half of our study respondents (57.1%) had a household size of 6 or more people. In the Ghanaian context, there are numerous societal responsibilities and pressures conferred on women with larger household sizes, as these women serve as the gatekeepers and managers of rural homes [9]. This can affect the time and resources the pregnant woman may require to start and sustain ANC and, in most cases, prioritize their farming or trading activities to provide for their families. Also, in some instances, those women who have experienced successful pregnancies may develop some level of confidence that they can survive the early period of pregnancy without any skilled assistance leading to late initiation [51,61,63].

Women who made fewer than four ANC visits were more likely to initiate their first ANC after the first trimester of pregnancy, consistent with other study findings [64,65]. This finding, though significant, could represent a cumulative deficit in care utilization rather than a predictive or causal factor. Evidence has shown that women who initiate early ANC have higher odds of receiving four or eight ANC contacts than those with late ANC initiation [8,51,66]. Higher ANC contacts and/or early ANC initiation are essential, as they ensure that pregnant women get all the required content and higher-quality ANC services at the right time [14,65]. Most women in rural communities have unplanned pregnancies and may not be aware of being pregnant until they feel unwell before visiting the health facility, which is usually late. Furthermore, other sociocultural and religious beliefs and misconceptions surrounding pregnancy within Ghanaian communities potentially discourage the use of orthodox healthcare services [56,57] which may lead to late or no ANC visits. Reportedly, low economic status, perception of no problem with pregnancy, and misinterpretation of recommendations from healthcare providers [51,67,68] could ignite late ANC initiation, leading to non-achievement of the recommended number of visits before delivery.

Home visit by health staff was independently associated with the time of ANC initiation. Home visits form a significant part of the daily activities of health staff in CHPS zones. This house-to-house visitation enables individuals and households to receive appropriate information and care at their doorstep throughout the RMNCH continuum of care [21,69]. Our study revealed that pregnant women were found to have higher odds of attending ANC late if they received a home visitation from health staff from the CHPS zone. This finding is comparable with a randomized control trial in Nigeria [70] but contradicts the results of a similar study by Kumbeni and his colleagues in the Upper East region of Ghana [46] which reported that women who received at least one home visit had higher odds of initiating early ANC and achieving at least eight ANC visits. The plausible reason for this finding may be due to the trust built between the pregnant woman and the healthcare professionals through home visit sessions [22,46]. These women misunderstand the services they received during the home visitation as the same as the facility care. Consequently, they become self-satisfied and do not find it appropriate to go to the health facility, especially when they feel nothing is wrong with them [51].

Monthly income offered significant protection for ANC initiation, as women with an average income between GHc 500–1000 had reduced odds of initiating late ANC services. This finding agrees with most studies in the SSA [58,59,71], which established a substantive relationship between women’s income level and initiation of ANC. High monthly wage offers these women enormous leverage to overcome late visitation [72,73]. Most Ghanaian women are unable to afford other indirect costs of accessing ANC (transportation, laboratory investigations, and other medications), despite the free maternal healthcare under the NHIS, making women who earn more income better placed to afford these services [71,74], and avail themselves for early ANC services. Nearly 77% of the study’s respondents were found to be self-employed, with most (39.4%) being farmers. Thus, their income is not regular and often not even close to the Ghanaian minimum wage due to the unstable economic situation in Ghana. The apparent financial constraint forces these women to adopt options to reduce expenditure, including delaying ANC initiation [75]. This gives credence to the importance of pro-poor policies that can augment the financial situation of these women and ultimately assist them in making appropriate reproductive health decisions.

### Strengths and limitations of the study

This is one of the few studies in Ghana that examined access to ANC by pregnant women in rural communities in the context of CHPS; thus, the findings provide valuable insights for policy strengthening. Moreover, the methodology used allowed for the gathering of primary data, which characterizes the uniqueness of the study setting. Restricting participants to only 12 months post-partum mothers preceding data collection reduced possible recall bias.

Nonetheless, the type of study design used may have limited the impact of the study due to the interpretation of the results as it could only allow for inferences but not causal relationships. Although there were efforts to limit information bias, the self-reporting used may have introduced recall and reporting biases. For instance, 40% of the mothers failed to disclose information on their monthly income, and others were not sure about their earnings which potentially weakened the power of the test and biased the results. Again, the nearly universal high knowledge scores created a ceiling effect, leaving too little variation to statistically predict ANC utilization. Additionally, since only mother-child pairs were used, information about mothers with adverse birth outcomes like perinatal deaths was not collected which might have affected study results. The use of the Euclidean distance to measure the distance of respondents to CHPS zones did not consider other topographical factors such as terrain. However, this method, though not sophisticated, has been an acceptable method for determining accessibility. On one hand, only functional CHPS zones were used as the unit of measure, even though some of the respondents were closer to other types of health facilities which may have limited the data as further studies may be required to bridge that knowledge gap. Again, the categories ‘CHPS’ and ‘clinic’ under place of ANC attendance may not have been distinguished by some respondents in the local dialect, as both are perceived locally as small health facility and could have led to some misclassification between these two categories, which we could not verify retrospectively.

Finally, during the statistical analysis, the use of a strict p < 0.05 cut-off in variable selection may have excluded moderately significant predictors (p < 0.20 or p < 0.25), potentially limiting the identification of relevant associations [38]. This decision was driven by previuos studies, sample size constraints and the need to maintain an appropriate case-to-variable ratio to avoid model instability and overfitting [39,76]. Also, sparse data in certain categorical variables (particularly marital staus, and place of delivery) with some categories containing fewer than five observations was accounted for during the bivariate analysis. Fisher’s exact test was applied when any expected cell counts fell below 5, in accordance with standard statistical practice. Moreover, their inclusion in the multivariable model might have also contributed to relatively wide confidence intervals, suggesting careful interpretation of associations. To enhance transparency, we have reported the model summary parameters (Nagelkerke R^2^ and Hosmer-Lemeshow test) to provide readers with an indication of overall model fit. We further suggest that future studies with larger sample sizes and more balanced distributions are recommended to validate these findings and strengthen the robustness of regression estimates.

## Conclusion

ANC coverage and early ANC initiation rate in the district were found to be high. Being unmarried, and home delivery were associated with at least one ANC visit. Independent correlates of late ANC initiation included larger household (family) size, fewer ANC contacts, home visitation by CHPS staff, and maternal average monthly income. Although a greater proportion of respondents received ANC services from CHPS zones, resided closer to CHPS zones, and had adequate knowledge about ANC and pregnancy, these expected plausible factors were not statistically associated with ANC attendance and time of ANC initiation. It therefore underscores the need for comprehensive and sustainable healthcare policies to address these gaps, creating inequities in access for women, especially those in rural communities.

The interaction of these given factors requires a multi-sectoral approach to mitigate them. The Government of Ghana through its relevant agencies including Ghana Health Service (GHS), should intensify the implementation of pro-poor policies such as Livelihood Empowerment Against Poverty (LEAP) which provides financial support for the poor, free maternal healthcare services, fertilizer subsidy for farmers, free education with more emphasis on girl education, among others, so that the livelihood of these vulnerable women in rural communities could be improved.

Additionally, the Ghana Ministry of Health (MoH) and stakeholders, including the Ministry of Education, Local Government Ministry, and other Non-Governmental Organizations should ensure more women in rural communities take advantage of the free education policy, thereby empowering them to make informed decisions on their reproductive rights.

The provision of accurate, appropriate, and timely information can potentially alter health-seeking behaviors in the district. Thus, the MoH and GHS should strengthen Behavior Change Communication strategies provided through health promotion and education. All possible avenues to share this information should be explored, including the use of community health management committees, community health volunteers, schools, community support groups, and community opinion leaders.

The MoH and GHS should also strengthen policies on home visits, including resourcing health facilities appropriately to gain the needed positive results. The content should be re-examined with a more innovative approach to providing the service and appropriate follow-up where necessary. Further qualitative studies need to be conducted to provide more insights into how these factors affect access to ANC and its impact on the individuals, households, and communities in the THLD district.

## Supporting information

S1 File De-identified datasets. De-identified datasets collected and analysed for the study (DTA).
(DTA)

S2 File GPS datasets. Anonymized GPS data for the study.
(XLSX)

## Abbreviations

ANC: Antenatal Care
CHN/O: Community Health Nurse/Officer
CHPS: Community-based Health Planning and Services
GHc: Ghana cedis
GHS: Ghana Health Service
GPS: Global Positioning System
MoH: Ministry of Health
PHC: Primary Health Care
RMNCH: Reproductive, Maternal, Newborn, and Child Health
SSA: Sub-Saharan Africa
THLD: Twifo Hemang Lower Denkyira
USD: United States of America Dollars
VIF: Variance Inflation Factor
WHO: World Health Organization
JH: John Hammond
SAG: Silas Adjei-Gyamfi
DBD: Doreen Brew Daniels
GKS: Godfred Kwabena Sarpong
HA: Hirotsugu Aiga
TA: Tsunoneri Aoki
